# Hyperphagia in rare melanocortin-4 receptor pathway diseases: therapeutic options and assessing treatment response

**DOI:** 10.1007/s11154-025-09984-3

**Published:** 2025-06-25

**Authors:** Jesús Argente, Karine Clément, Jessica Duis, I. Sadaf Farooqi, Peter Kühnen, Jennifer L. Miller, Christian L. Roth, Erica van den Akker

**Affiliations:** 1 Hospital Infantil Universitario Niño Jesús, Madrid, Spain; 2 Universidad Autónoma de Madrid, Madrid, Spain; 3 CIBER Fisiopatología y Nutrición (CIBEROBN), Instituto de Salud Carlos III & IMDEA Food Institute, Madrid, Spain; 4 Sorbonne University, Inserm, Nutrition and Obesities; Systemic Approaches, NutriOmique Research Group, Paris, France; 5 Nutrition Department, Assistance Publique-Hôpitaux de Paris, Pitié-Salpêtrière Hospital, Paris, France; 6 RareDiseaseDoc, LLC, Aurora, CO USA; 7 Institute of Metabolic Science and NIHR Cambridge Biomedical Research Centre, University of Cambridge, Cambridge, UK; 8 Department of Pediatric Endocrinology and Diabetology, Charité-Universitätsmedizin Berlin, Corporate Member of Freie Universität Berlin Und Humboldt-Universität Zu Berlin, Berlin, Germany; 9 Pediatric Endocrinology, Department of Pediatrics, College of Medicine, University of Florida, Gainesville, FL USA; 10 Seattle Children’s Research Institute, Seattle, WA USA; 11 Division of Endocrinology, Department of Pediatrics, University of Washington, Seattle, WA USA; 12 Erasmus University Medical Center, Rotterdam, The Netherlands

**Keywords:** Monogenic obesity, Syndromic obesity, Hypothalamic obesity, Leptin-melanocortin system, MC4R

## Abstract

Hyperphagia is a hallmark of both congenital and acquired rare melanocortin-4 receptor (MC4R) pathway diseases. Currently, the medical community has no standard treatment guidelines or approach to establishing treatment benefit. This narrative review discusses current understandings of the pathophysiology, burden, and treatment of hyperphagia and summarizes findings from a systematic literature review of validated instruments for assessing the response to hyperphagia treatment. Hyperphagia can result from dysfunction within, or damage impacting, hypothalamic pathways including the MC4R pathway, a key regulator of energy balance. The burden of hyperphagia is substantial, with negative effects experienced across physiologic, emotional, and social domains. Approaches for hyperphagia management include environmental control, lifestyle intervention, pharmacotherapy, neurocognitive approaches, and neurostimulation. There are varied approaches to determine treatment response; however, standard methodology has not been determined and largely relies on questionnaires. Studies of rare MC4R pathway diseases have improved understanding of the etiology of hyperphagia and established the need for indication-specific treatment. Targeted treatments are limited, and methods for determining treatment efficacy are varied. There is a need for consensus guidelines to establish a standard approach for the management of hyperphagia and related assessment of treatment response to improve patient morbidity.

## Introduction

Hyperphagia is a hallmark of congenital or acquired rare melanocortin-4 receptor (MC4R) diseases. Evidence supports a key role of the MC4R pathway in the etiology of hyperphagia, given that this pathway is a key regulator of energy balance [1–9]. Hyperphagia is a pathologic, insatiable hunger (ie, increased drive to eat) accompanied by abnormal food-seeking behaviors and impaired satiety [10, 11]. However, an international consensus on the definition and differentiation from overeating behaviors is lacking because there is no uniform instrument to measure the symptoms and severity of hyperphagia, which can vary across patients [9–15]. Investigations into the pathophysiology of genetic and acquired hypothalamic obesities (HOs) have shown that the mechanisms underlying hyperphagia extend, in part, from dysfunction or damage that impacts the MC4R pathway and have informed development of new pharmacotherapies in these patient populations [2, 5, 9, 16, 17].

According to current clinical practice guidelines for obesity, the presence of hyperphagia and early-onset, severe obesity (ie, ≥ 120% of the body mass index [BMI] 95th percentile occurring before the age of 5 years) constitutes an indication for genetic testing, which is critical to determine appropriate disease management [3, 18]. Hyperphagia causes pervasive negative consequences for patients and their caregivers, including weight gain, and impacts relationships and productivity, which in some cases can be mitigated by targeted treatment [12, 14]. There are no standard guidelines for the treatment of hyperphagia. Patients may receive supportive care including environmental control and pharmacotherapies; however, without addressing the underlying cause of hyperphagia, the efficacy of symptom management is often limited [5, 12, 19].

This review aims to characterize hyperphagia and discuss currently available approaches for its management and assessment through 4 key topics: (1) pathophysiology of hyperphagia and MC4R signaling, (2) the impact and burden, (3) evidence for current management approaches, and (4) tools that can be used to measure change in hyperphagia following treatment.

## Pathophysiology of hyperphagia: impaired leptin-melanocortin signaling

Energy homeostasis is a tightly regulated process that involves key neurohormonal systems underlying hunger (ie, the physiologic signal that drives eating), food reward (ie, the cognitive or sensory experience of the desire to eat), satiety (ie, the physiologic state in which further eating is inhibited by fullness), and energy expenditure [20–24]. Chronic destabilization of any of these processes results in weight gain, which can increase morbidity and decrease longevity [22, 25, 26]. Overeating behaviors are not restricted to patients with overweight or obesity; although hunger is often subjective and a usual amount of food intake difficult to define [27, 28], some behaviors can range from normal (over indulgence or feasting) to abnormal and from occasional to constant [11, 29]. It is important to differentiate hyperphagia from other overeating behaviors such as emotional overeating, hedonic overeating, and binge eating, which are reviewed elsewhere [11, 15, 29]. Notably, although excessive energy intake is common among all overeating behaviors, hyperphagia is distinct in terms of being the most extreme with respect to frequency, duration, severity, and food-seeking behaviors [15].

**Fig. 1:**
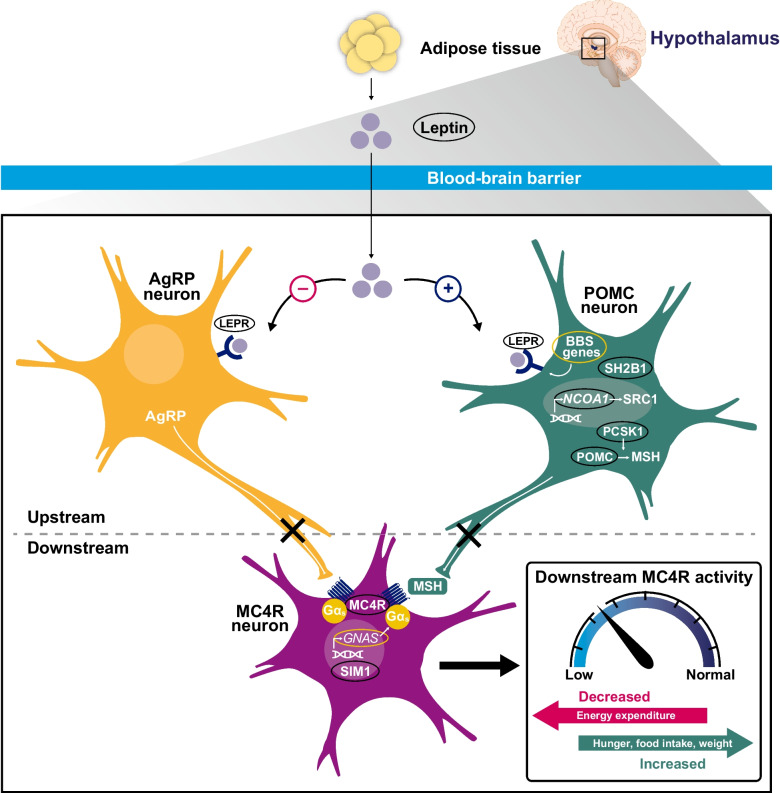
MC4R pathway regulation of energy balance. AgRP, agouti-related peptide; BBS, Bardet-Biedl syndrome; Gα_s_, stimulatory G-protein alpha subunit; LEPR, leptin receptor; MC4R, melanocortin-4 receptor; MSH, melanocyte-stimulating hormone; PCSK1, proprotein convertase subtilisin/kexin type 1; POMC, proopiomelanocortin; SCR1, steroid receptor coactivator 1; SIM1, single-minded homolog 1; SH2B1, SH2B adaptor protein 1

Delineation of the neurohormonal systems that regulate food intake, hunger, and satiety under normal conditions provides crucial insight into the pathophysiology of hyperphagia. Decades of research in humans and in animal models have identified the leptin-melanocortin system—also referred to as the MC4R pathway—as a key regulator of energy balance (ie, energy intake vs energy expenditure; Fig. 1) [22]. Within the leptin-melanocortin system, peripheral hunger and satiety hormones include leptin, insulin, ghrelin, glucagon-like peptide-1 (GLP-1), cholecystokinin, and peptide YY, among others. These factors can alter neuronal signaling in the hypothalamus and other key brain regions (eg, nucleus tractus solitarius, ventral tegmental area, nucleus accumbens), which in turn promotes increased or decreased energy intake [22, 24, 30–33]. Specifically within the hypothalamus, 2 distinct neuronal populations in the arcuate nucleus (ARC) of the hypothalamus project to MC4R-expressing neurons of the paraventricular nucleus of the hypothalamus and exert opposing influences on food intake [22, 24]. In a fasted state, leptin binds to leptin receptors (LEPRs) on ARC neurons that coexpress agouti-related peptide (AgRP), neuropeptide Y (NPY), and γ-aminobutyric acid (GABA), thereby increasing production of the MC4R antagonists AgRP and NPY that results in stimulating food intake [2, 22, 24]. In contrast, in a fed state, leptin secreted from adipose tissue binds to LEPRs on AgRP-expressing ARC neurons, thereby suppressing AgRP and NPY production, disinhibiting MC4R, and decreasing food intake [2, 9]. Also in a fed state, leptin and insulin bind to their receptors on proopiomelanocortin (POMC)–expressing neurons of the ARC, leading to increased production of POMC, which is processed and cleaved into α–melanocyte-stimulating hormone (α-MSH) and β-MSH by proprotein convertase 1/3 (PC1/3; encoded by *PCSK1*) [2, 9, 30]. Activation of MC4R by α-/β-MSH decreases food intake, an effect that may be enhanced by downstream MC4R signaling within extrahypothalamic brain regions that respond to gastrointestinal factors secreted following ingestion [2, 32–34].

Rare variants in key genes, gene families, or chromosomal regions that affect the MC4R pathway, including *LEP*, *LEPR*, *MC4R*, *MRAP2*, *NCOA1* (encodes steroid receptor coactivator 1), *PCSK1*, *POMC*, *SH2B1*, *SEMA3A-G*, *SIM1*, genes associated with Bardet-Biedl syndrome (BBS), and the 16p11.2 chromosomal region, can disrupt MC4R pathway signaling and are associated with hyperphagia and/or early-onset, severe obesity [2, 5, 7, 8, 35–37]. Some evidence also suggests chromosome 15q11-13 deletion common to Prader-Willi syndrome (PWS) may also impact the MC4R pathway through deletion of *MAGEL2* or *SNORD116*, which alter leptin receptor signaling and expression of serotonin 2 C receptors involved in activation of POMC neurons, respectively [11, 38–42]. Additionally, damage to the hypothalamus from intracranial tumors, trauma, irradiation, or surgery can potentially impact MC4R signaling. This reduction in activation of the MC4R pathway can subsequently reduce sympathetic tone, consequently leading to hyperphagia in patients with HO [1, 43–47]. Notably, the severity of hyperphagia may be associated with the degree of impairment to the leptin-melanocortin system. As an example, genetic variants or hypothalamic damage that affects anorexigenic signaling (ie, signaling that decreases food intake under normal conditions) via both POMC and AgRP ARC neurons may be associated with more severe hyperphagia than those that affect only 1 anorexigenic signaling pathway [2, 48]. Additionally, different variants within the same gene may also result in similar variability in the severity of hyperphagia [48].

## Clinical and quality of life impacts of hyperphagia

Natural history studies, patient and caregiver surveys, and clinical trials of patients with rare MC4R pathway diseases associated with hyperphagia and obesity have characterized both the presentation and burden of hyperphagia [11, 12, 14, 19, 49]. In the context of MC4R pathway diseases, hyperphagia can be defined as a pathologic, insatiable hunger that is accompanied by abnormal food-seeking behaviors and impaired satiety, promoting increased food intake. Briefly, hyperphagia can be identified by symptoms associated with an extreme, insatiable drive to consume food, including food-seeking behaviors and related anxiety or distress caused by a severe preoccupation with food or by limited access to food [10, 11, 19]. The impact of such behaviors on patient health and quality of life (QOL) are relatively proportional to the degree of impairment [12, 14, 29, 48].

Owing to a chronic imbalance between energy intake and expenditure, hyperphagia may contribute to development of severe obesity in individuals with rare MC4R pathway diseases [1, 5, 11, 22]. Furthermore, patients with these diseases typically do not achieve sustained weight loss and can continue to gain weight with traditional weight management strategies (ie, lifestyle modification, traditional antiobesity medications, metabolic and bariatric surgery [MBS]) because these treatments do not address the pathophysiology of hyperphagia [1, 17, 50–56]. Critically, challenges with weight management increase the risk and severity of obesity, obesity-related comorbidities, and premature death [25, 57–59], which may be further heightened in patients with rare MC4R pathway diseases [1, 3, 57, 60–63].

**Fig. 2:**
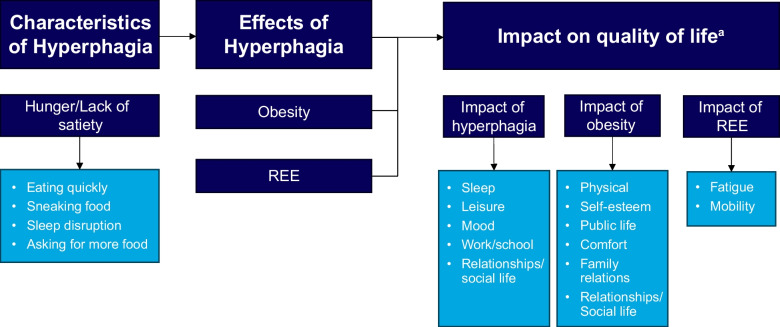
Impact of hyperphagia on quality of life. REE, resting energy expenditure. ^a^The impact on quality of life across the listed domains have been assessed with age-appropriate questionnaires including Impacts of Hyperphagia, Impact of Weight on Quality of life, EQ-5D, 36-item Short Form Health Survey (SF-36), Work Productivity and Activity Impairment (WPAI), and Pediatric Quality of Life Inventory (PedsQL)

Beyond contributing to development of obesity and related comorbidities, the quality-of-life burden of hyperphagia is substantial, with both patients and their caregivers reporting serious social and emotional consequences (Fig. 2) [64, 65]. Specifically, in a multicountry survey that assessed the burden of hyperphagia on patients and caregivers of patients with BBS, > 50% of caregivers reported that hyperphagia had at least a moderate negative impact on the patient’s sleep, mood or emotions, school or work performance, leisure, and familial relationships [19]. In qualitative interviews, patients and caregivers of patients with POMC deficiency caused by biallelic variants in *POMC* or *PCSK1*, LEPR deficiency caused by biallelic variants in *LEPR*, or BBS reported that hyperphagia was always or nearly always present, led to negative emotions such as guilt and failure, and negatively affected friendships, family dynamics, and productivity at school or work [12, 14]. Considering the burden of hyperphagia and relationship between hyperphagia, obesity, and obesity-related comorbidities and mortality, effective hyperphagia treatment is an urgent need in this patient population.

## Approaches for managing hyperphagia

Despite the substantial impact of hyperphagia, no standard guidelines have been established for this condition, and the strategies used for managing symptoms of hyperphagia include nonpharmacologic and pharmacologic interventions. A systematic literature review (SLR) was undertaken to explore current hyperphagia assessments or methods to quantify hyperphagia [15]. Publications identified in the SLR were further reviewed to identify approaches studied for managing hyperphagia. The SLR identified 1628 records, 1515 of which were excluded after title and abstract screening; the remaining 101 were reviewed, and 78 met the inclusion criteria. An additional case report search and additionally recommended articles included 23 case studies or series that included 53 patients with POMC deficiency, LEPR deficiency, MC4R deficiency, BBS, or HO. From these findings, a subset of identified publications described hyperphagia or hyperphagia-related outcomes after behavioral or lifestyle interventions, pharmacotherapy, MBS, or experimental treatment (eg, noninvasive brain stimulation, deep brain stimulation).

**Fig. 3:**
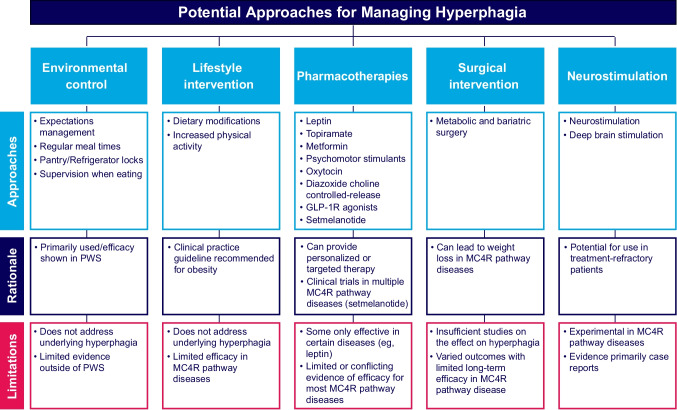
Current potential approaches for managing hyperphagia. GLP-1R, glucagon-like peptide-1; MC4R, melanocortin-4 receptor; PWS, Prader-Willi syndrome

Publications summarized here were identified from this SLR as related to hyperphagia management with the further inclusion of other articles identified by the authors that describe hyperphagia treatment. Additionally, to provide a comprehensive review, case reports identified through iterative searches in Ovid Embase and PubMed were also added to this work. Briefly, the terms “melanocortin 4 receptor,” “MC4R,” “leptin receptor,” “LEPR,” “proopiomelanocortin,” “POMC,” “Bardet-Biedl syndrome,” “BBS,” and/or “PCSK1” were used in combination with “obesity” with the case reports filter applied to identify studies reporting on patients with hyperphagia in populations of interest. The titles and abstracts from these searches were screened to identify case reports describing any intervention for hyperphagia, and full texts of identified studies were further reviewed to ensure relevance to this work. Findings are summarized herein (Fig. 3).

### Environmental control

Because hyperphagia is commonly associated with PWS, detailed study of the clinical course of hyperphagia in PWS has led to the development of standard environmental control for managing hyperphagia. Studies on the clinical course and impact of hyperphagia have also been performed for rare MC4R pathway diseases [11, 12, 19]. The key aims of behavioral management are to limit food intake and mitigate disappointment or distress resulting from unmet expectations of food consumption [11, 66]. Environmental control may include managing expectations of what is available to eat, setting regular meal times, controlling access to food via pantry or refrigerator locks, and supervision to prevent overeating [11, 19, 66]. Notably, patients with PWS often have developmental and behavioral characteristics [67]. For these patients, behavioral management together with multidisciplinary care to address other complex features (eg, endocrine abnormalities) can help to reduce BMI to within the normal range [67]. A limited number of studies have assessed these types of behavioral interventions in other diseases associated with hyperphagia. In patients with MC4R pathway diseases, being informed of hyperphagia and its impacts can improve patient and caregiver burden and associated stigma, although symptoms can persist without targeted intervention [65, 68, 69]. Additional evidence is needed to indicate whether similar early environmental control by caregivers can aid in regulating hyperphagia behaviors and lead to effective weight management in other causes of obesity.

### Lifestyle intervention

Clinical practice guidelines recommend lifestyle intervention (eg, dietary modifications, increased physical activity) as an initial approach for weight management in patients with obesity [70]. However, these interventions often focus on managing obesity itself and not the underlying hyperphagia. Further, studies investigating the efficacy of lifestyle interventions for treatment of rare MC4R pathway diseases are limited, show variable weight outcomes, and do not report changes in hyperphagia. For example, case reports, natural history studies, and medical histories of patients with rare MC4R pathway diseases describe multiple unsuccessful attempts to lose weight through lifestyle intervention [53–55, 71]. Additionally, of 4 studies identified wherein patients with rare MC4R pathway diseases underwent lifestyle intervention, only 1 case series of 2 patients with BBS reported sustained weight loss (− 11 and − 12 kg/m^2^ BMI change in the first and second patient, respectively, from baseline to Year 2 of a 1400-kcal/day and 0.6-g protein/kg/day diet), and none assessed changes in hyperphagia [72–75]. In a Danish cohort of 30 children with *MC4R* variants, lifestyle intervention with a multidisciplinary care team did not improve BMI after a median of 1 year (range, 0.5 to 4.0 years) of treatment [75]. A

German cohort of 9 children with *MC4R* variants did report that patients lost weight with 1 year of lifestyle intervention; however, these results were not maintained after 1 year without intervention [74].

### Pharmacotherapies

Pharmacotherapies commonly used for the management of hyperphagia include leptin, topiramate, metformin, psychomotor stimulants, oxytocin, GLP-1 receptor (GLP-1R) agonists, and the MC4R agonist setmelanotide, all of which are hypothesized to exert weight loss effects, at least in part, through changes in hunger, appetite, food reward, or food-liking (ie, hedonic impacts of food reward signaling) [76].

#### Leptin

Patients with monogenic obesity due to homozygous *LEP* variants can be treated with recombinant leptin to correct the deficiency [5, 77]. This treatment can lead to weight loss and reduced food intake [77–80]. In 1 report of 3 children with leptin deficiency, leptin treatment resulted in a 45%−84% reduction in energy intake after 2 months [77]. Similar results have been observed in adult patients. A Turkish cohort of 3 adults with leptin deficiency had marked weight loss and food intake reduction after 4–6 months of leptin treatment, with a 49% decrease in mean daily caloric intake after 2 weeks [79]. Leptin treatment is not effective in patients with genetic variants affecting or physical damage to the MC4R pathway at or downstream of LEPR because impaired LEPR or key pathway components such as POMC or PC1/3 inhibit pathway activation from leptin signaling [5].

#### Topiramate

Topiramate is indicated for the treatment of epilepsy and migraine but can exert weight loss effects by promoting increased satiety and reduced caloric intake through increased GABAergic signaling, decreased glutamatergic signaling, and reduced NPY levels [81–83]. Three studies examining the efficacy of topiramate in patients with POMC deficiency or PWS were identified [84–86]. A case study of a pediatric patient with POMC deficiency reported improved hyperphagia with topiramate treatment (duration of 3 years) that was reversed when topiramate was discontinued [84].

Additionally, an 8-week, randomized, double-blind, placebo-controlled trial of 62 patients with PWS showed significantly larger reductions in hyperphagia from baseline in patients treated with topiramate than in those treated with placebo [85]. Notably, however, sedative effects or psychomotor slowdowns, biologic modifications in hepatic function, and hyperammonemia were reported in 28.6% of patients treated with topiramate, consistent with reports of increased somnolence in patients with PWS treated with topiramate [85, 86]. In contrast to studies showing improved hyperphagia, a case series showed continued or worsened hyperphagia in 2 of 5 patients with PWS who received topiramate [86].

#### Metformin

Metformin is widely used for treating type 2 diabetes and is reported to suppress appetite, potentially by activating the integrated stress response pathway, which leads to secretion of growth differentiation factor 15 and improved glycemic regulation [87]. In a case report of a child with POMC deficiency, the patient experienced a 2.0-kg/m^2^ reduction in BMI after 2 years of metformin treatment. Although hyperphagia was not directly assessed, the authors posited that curbed weight gain was related to the effect of metformin on hyperphagia [88]. A natural history study of patients with POMC or LEPR deficiency and medical histories collected during a Phase 2 trial of setmelanotide in patients with HO revealed that 1 of 8 patients with LEPR deficiency and 2 of 18 with HO had previously tried unsuccessfully to lose weight with metformin, and hyperphagia change with metformin was not reported [53, 71]. A pilot study of 21 patients with PWS and 10 with early-onset obesity reported significant reductions in total hyperphagia scores for both groups after variable lengths of time receiving metformin (range, 6 months to 3 years) [89].

#### Psychomotor stimulants

Psychomotor stimulants (eg, dextroamphetamine) are typically used to treat attention-deficit/hyperactivity disorders, but mild appetite suppression is a commonly reported adverse event [90]. Reduced appetite and weight loss with psychomotor stimulants are thought to result from increased sympathetic tone and dopamine reuptake inhibition in brain regions that regulate hedonic eating (ie, eating for pleasure) [91, 92]. In a retrospective case series, 6 of 7 patients with HO experienced reduced or stabilized BMI after treatment with dextroamphetamine (treatment duration range, 1.0–4.5 years); changes in hyperphagia were not reported for these patients, but those patients who did not experience sustained BMI reduction regained weight during the second year of treatment because of reoccurrence of hyperphagia [93]. Another retrospective study of patients with HO also found reductions in BMI Z scores over 12 months of treatment with dextroamphetamine [94]. Of 17 patients evaluated, 10 reported improved satiety, 8 reported improvements in hyperphagia behaviors, and 5 reported increased activity levels. The effects of dextroamphetamine treatment on BMI and hyperphagia were reduced over time (mean treatment duration of 23.7 months).

Additionally, historic reports of treatment with dextroamphetamine have been identified in studies of patients receiving other pharmacotherapy for weight management. In a natural history study of patients with POMC or LEPR deficiency, 2 of 8 patients with LEPR deficiency reported previous unsuccessful attempts to lose weight with methylphenidate or dextroamphetamine, although the impact on hyperphagia was not reported [53]. Similarly, medical histories of 18 patients with HO enrolled in a Phase 2 trial of setmelanotide showed previous unsuccessful attempts to lose weight (hyperphagia outcomes not reported) with methylphenidate in 2 patients and dextroamphetamine in 1 patient [71].

#### Oxytocin

In addition to its functions related to social behavior and lactation, the endogenous neuropeptide oxytocin is involved in regulation of eating behaviors and satiation [95]. Medical histories collected from 18 patients with HO during a Phase 2 trial of setmelanotide revealed that 3 patients had attempted unsuccessfully to lose weight with oxytocin or naltrexone, although the effect on hyperphagia was not reported [71]. No other identified studies investigated the efficacy of oxytocin for rare MC4R pathway diseases; however, the SLR identified 4 clinical trials that investigated the efficacy of intranasal oxytocin for improving satiety in patients with PWS, with varied outcomes. While most studies reported limited to no difference in hyperphagia, 1 placebo-controlled study found treatment with oxytocin over 8 weeks decreased hyperphagia in pediatric patients aged < 11 years [96–99]. Currently, there is limited evidence supporting the potential use of oxytocin in MC4R pathway diseases, and oxytocin is not approved for the treatment of hunger or hyperphagia.

#### Diazoxide choline extended-release tablets

Diazoxide choline controlled-release (DDCR) tablets were recently approved by the US Food and Drug Administration as the first treatment for hyperphagia in patients with PWS. DDCR targets adenosine triphosphate–sensitive potassium channels in NPY/AgRP/GABA neurons to reduce NPY secretion, a driver of hyperphagia in PWS [100]. In the Phase 3, randomized, double-blind DESTINY PWS trial, DDCR treatment was associated with significant hyperphagia reductions compared with placebo in patients who had severe hyperphagia at baseline, although no significant differences in hyperphagia were observed between DDCR and placebo in the overall study population [101]. In a trial including a randomized withdrawal period, patients with PWS receiving placebo had worsening hyperphagia as well as increased weight, BMI, and BMI Z score following DDCR withdrawal compared with patients remaining on DDCR after 16 weeks [102]. To date, the authors are not aware of any published data evaluating the efficacy and safety of DDCR for hyperphagia in MC4R pathway diseases.

#### GLP-1R agonists

Glucagon-like peptide-1 receptor agonists have led to significant weight reduction in patients with overweight or obesity [103, 104]. Whereas weight loss with GLP-1R agonists occurs primarily as a result of decelerated gastric emptying, reduced appetite likely occurs via both suppression of AgRP neuronal activity and increased satiety due to activation of brainstem receptors [16, 105–110]. Six identified publications reported hyperphagia-related outcomes in patients with HO or PWS who were treated with GLP-1R agonists [111–116]; 1 case study of a patient with BBS also reported BMI reduction with GLP-1R agonist treatment, but change in hyperphagia was not reported [117]. Three publications reported the effects of exenatide and semaglutide in patients with HO. In 1 case study, a patient with HO secondary to craniopharyngioma exhibited BMI stabilization, increased satiety, and improved QOL with exenatide [114].

Additionally, a 52-week pilot study of exenatide in 10 patients with HO secondary to craniopharyngioma (n = 6), astrocytoma (n = 1), or other pituitary or hypothalamic tumor (n = 3) showed a significant decrease in reported energy intake, as assessed by a 24-h dietitian-led food recall, from baseline to Week 52 of treatment (mean [SD] intake, 7837.8 [2796.6] vs 6258.4 [1970.7] kJ; *P* = 0.027). This change in energy intake was significantly correlated with weight change (R^2^ = 0.70; *P* = 0.009), although buffet meal food consumption did not change with treatment [115]. A case study of a patient with HO secondary to craniopharyngioma who was treated with semaglutide and metformin showed reduced weight, improved satiety, increased control over food intake, and improved mood and QOL after 6 months of treatment [116]. Although not identified in our SLR, an additional randomized, double-blind, placebo-controlled clinical trial for Examination of Energy Balance and Weight loss in Hypothalamic Obesity (ECHO) was included [118]. Patients with suprasellar tumors who received 36 weeks of once-weekly injections of 2-mg extended-release exenatide showed a decrease in food intake with ad libitum study meals and an increase in fullness. There were no significant differences in changes of BMI in patients treated with exenatide versus placebo; however, there was a moderate significant change in percent fat mass between groups favoring exenatide treatment (mean difference, − 3.1 kg; *P* = 0.04). While there was inconsistency in the response, parents reported children receiving exenatide ate more slowly. In comparison to reports of improved satiety and reduced energy intake with GLP-1R agonist treatment, 6 of 18 patients with HO who enrolled in a Phase 2 trial of setmelanotide reported having previous unsuccessful weight loss attempts with exenatide (n = 3), liraglutide (n = 2), or semaglutide (n = 1) [71]. While some studies of GLP-1R agonists reported moderate improvement of hyperphagia in pediatric populations with PWS using the Hyperphagia Questionnaire, an SLR of 10 publications involving 23 patients with PWS treated with the GLP-1R agonists exenatide and liraglutide (range, 14 weeks to 4 years) identified evidence gaps and substantial heterogeneity in administration in this patient population [111–113].

#### Setmelanotide

Because MC4R pathway dysfunction is implicated in the pathophysiology of hyperphagia, MC4R agonists such as setmelanotide were hypothesized to reduce hunger and weight by restoring pathway signaling [7, 16, 43]. Setmelanotide is approved for weight and/or hunger management in patients aged ≥ 6 years with POMC deficiency (including biallelic *PCSK1* variants), LEPR deficiency, and BBS [119–121]. Ten identified publications reported effects of setmelanotide on weight, hunger, or health-related QOL (HRQOL) in patients with rare MC4R pathway diseases associated with hyperphagia and obesity [12–14, 28, 71, 122–126].

Two 52-week, Phase 3 trials showed significant mean [SD] percent reductions from baseline in hunger scores (assessed by a Likert scale where 0 = not hungry at all and 10 = hungriest possible; see Daily Hunger Questionnaire below) after ~ 1 year of treatment with setmelanotide in patients with POMC deficiency (− 27.1% [28.1%]; *P* = 0.0005; n = 10) or LEPR deficiency (− 43.7% [23.7%]; *P* < 0.0001; n = 11) [124]. These changes were accompanied by significant reductions in weight and age-appropriate weight-related parameters (ie, BMI for patients aged ≥ 18 years and BMI Z score for patients aged < 18 years) [124]. Similarly, in another Phase 3 trial, 62.5% of patients with BBS or Alström syndrome aged ≥ 12 years without cognitive impairment achieved a ≥ 25% decrease from baseline in hunger score (assessed by the same scale) after 52 weeks of setmelanotide treatment (mean [SD] percent change, − 30.9% [24.7%; *P* < 0.0001]; n = 31). Hunger improvement was accompanied by clinically meaningful reductions in age-appropriate weight-related parameters among patients with BBS, although weight results were inconclusive for the small sample of patients with Alström syndrome (n = 6) [13].

Secondary analyses of these trials included assessments of HRQOL and qualitative interviews to characterize hyperphagia burden and its alleviation with setmelanotide [12, 14, 28, 125]. In patients with POMC or LEPR deficiency, most evaluable patients showed HRQOL impairment at baseline but experienced clinically meaningful improvement after 52 weeks of setmelanotide treatment [125]. The authors hypothesized that improved HRQOL was related to reduced hunger and weight with setmelanotide [125]. Similarly, most evaluable patients with BBS had impaired HRQOL at baseline that improved with setmelanotide, and all patients without baseline impairment either improved or maintained nonimpaired status after 52 weeks of setmelanotide treatment, concomitant with meaningful within-patient changes in hunger [28]. In qualitative interviews, patients with POMC deficiency, LEPR deficiency, or BBS (including caregivers of patients with BBS) who completed Phase 3 trials of setmelanotide reported that before treatment, hyperphagia was always or nearly always present and associated with substantial social and emotional consequences [12, 14]. All participants reported substantial improvements in hyperphagia with setmelanotide treatment and described them as highly meaningful.

Setmelanotide has also been associated with weight and hunger improvement in Phase 2 trials of patients with HO; *POMC*, *PCSK1*, or *LEPR* heterozygous variants; steroid receptor coactivator 1 deficiency; SH2B adaptor protein 1 deficiency; and 16p11.2 chromosomal deletion [71, 122, 123, 126]. Hunger improvements were also observed in patients with Smith-Magenis syndrome receiving setmelanotide, although no benefits for weight reduction were seen at the end of 4 months of treatment [127]. In a 4-week, Phase 2 study in PWS, setmelanotide showed no difference from placebo in weight change and nonsignificant reductions in hyperphagia questionnaire scores; future research is needed to elucidate the potential efficacy of setmelanotide in this population [128].

### Metabolic and bariatric surgery

Clinical practice guidelines recommend MBS in patients with severe obesity who do not experience weight loss with lifestyle intervention or pharmacotherapy [70]. In addition to weight loss, reduced hunger or appetite are commonly reported after MBS and are hypothesized to result from changes in gut hormone secretion and activity in peripheral and central targets of those hormones (eg, GLP-1Rs in the ARC) [129, 130]. Critically, however, MBS does not directly target hyperphagia pathophysiology, and it has been suggested MBS should be reserved as a final option to manage severe obesity-related comorbidities in patients with hyperphagia due to genetic obesity [131]. Accordingly, 12 publications reporting weight and/or hyperphagia outcomes of MBS in patients with rare genetic causes of obesity or HO were identified, with varied outcomes and limited long-term success [50–52, 54, 132–139].

Seven publications reported outcomes of MBS in patients with obesity caused by rare variants in *LEPR*, *MC4R*, *NCOA1*, *PCSK1*, *POMC*, *SH2B1*, or *SIM1* [51, 52, 54, 133, 135, 136, 140]. Although some initial weight loss was reported in studies that used MBS for treatment of MC4R-related deficiencies, this weight loss was not sustained, and the presentation of hyperphagia in MC4R deficiencies may vary from those with variants upstream of MC4R [51, 52, 54, 136]. Most studies examined various MBS methodologies in patients with *MC4R* variants. One case study of a patient with 2 heterozygous MC4R mutations who underwent laparoscopic truncal vagotomy and laparoscopic adjustable gastric banding reported initial weight loss (− 11.6 kg) at Month 4 followed by regain of all preoperative weight by Month 12 and persistence of insatiable hunger [133]. In contrast, a case study of a patient with a heterozygous *MC4R* variant reported substantial weight loss (− 75.8% excess weight loss) and appetite suppression 5 years after Roux-en-Y gastric bypass (RYGB) [135]. In a family with *MC4R* variants (n = 4 homozygous; n = 3 heterozygous), MBS, including laparoscopic adjustable gastric banding, laparoscopic sleeve gastrectomy, and laparoscopic RYGB did not significantly reduce BMI, and 3 of 4 patients with homozygous variants regained preoperative weight 2 to 5 years after surgery; changes in hyperphagia were not reported [136]. Conversely, a study of 62 patients with heterozygous *MC4R* variants found weight loss was similar between carriers and noncarriers following RYGB, suggesting a single copy of the gene is sufficient to achieve weight loss with MBS [140].

One case series reported results of MBS in 2 patients with LEPR deficiency (sleeve gastrectomy, n = 1; gastric banding, n = 1); in both cases, MBS led to initial weight loss but eventual weight regain or complications [54]. A retrospective analysis of a broad population of patients with LEPR, POMC, or MC4R deficiency did report initial weight loss with gastric banding in 2 patients with LEPR deficiency, with sleeve gastrectomy in 1 patient each with LEPR, POMC, and MC4R deficiency, and with gastric bypass in 2 patients with LEPR deficiency and 1 patient with POMC deficiency; critically, severe hyperphagia persisted in all patients after MBS [52]. A case–control study of 50 patients with heterozygous variants in *LEPR, PCSK1, POMC, SH2B1, SRC1, MC4R*, or *SIM1*, carriers demonstrated higher maximum weight regain 15 years after RYBG compared with noncarriers [51].

Five publications reported MBS outcomes in patients with BBS. Two studies of patients with gastric bypass did not include the impact of treatment on hyperphagia, but they do report clinically meaningful initial weight loss with varied reports on weight regain [132, 134]. There were 3 case studies that examined patients who underwent sleeve gastrectomy [50, 137, 138], 2 of which reported reduced hunger, food intake, or appetite [50, 138]. One case series reported outcomes of Roux-en-Y gastric bypass in 3 patients with HO. All patients showed transient weight loss, and 1 patient who achieved − 44.6% total body weight loss at nadir and − 24.6% total body weight loss at 11.5 years reported reduced hunger at both 18 months and 14 years after surgery [139].

### Neurostimulation

Neurostimulation, including noninvasive and deep brain stimulation, has been proposed as a potential therapeutic option for treatment-refractory patients with severe obesity because of its ability to modulate activity in key brain regions associated with food reward or executive control [141, 142]. Two identified publications reported the effects of neurostimulation on hyperphagia in patients with HO or PWS [143, 144]. In 1 case study, deep brain stimulation of the nucleus accumbens—a brain region strongly implicated in motivated behavior and food reward—led to decreased preoccupation with food in a patient with HO secondary to craniopharyngioma, although the report did not specify duration of treatment or treatment effects [76, 143]. Lastly, in a pilot study, 10 patients with PWS were administered transcranial direct current stimulation (2.0 mA, 30 min once daily, 5 consecutive days) or sham stimulation over the dorsolateral prefrontal cortex—a brain region involved in executive control [142, 144]. The authors reported a significant decrease from baseline in Hyperphagia Questionnaire total scores on Day 15 (ie, 10 days after the final stimulation session) with active stimulation, as well as significantly lower food craving (assessed by a visual numerical scale [0 = not hungry at all and 10 = very hungry]) for active compared with sham stimulation on Day 15 [144].

## Assessment of treatment response

There are a variety of methods that can be used to assess the impact of treatment on hyperphagia including ad libitum meals studies and visual analog scales, both of which can be combined with functional magnetic resonance imaging (fMRI), as well as validated questionnaires [10, 11, 118, 145]. Generally, these methods can establish the symptoms and characteristics of hyperphagia and their related changes after intervention while ad libitum meals can provide additional quantitative details of energy intake [10] and fMRI can identify changes in brain networks or brain activity within patients or between patient groups [10, 11, 145–148]. Although these techniques can provide valuable insights, they each convey their own challenges. The ethics of ad libitum meals has been questioned because it can be dangerous to provide unrestricted food access to patients with hyperphagia, particularly those with PWS [10]. In the case of fMRI studies, the logistics and accessibility of scanners may be impacted by the necessity to use machines with varying bore diameters to accommodate data acquisition in patients with severe obesity scanners [149]. Additionally, while studies involving ad libitum meals or fMRI may be appropriate for use in clinical research, it may be more challenging to implement such methods in a standard clinical setting.

Thus, in order to quantify treatment efficacy in patients with hyperphagia, it is necessary to use questionnaires validated for specific underlying pathologies [11]. Whereas several validated, indication-specific questionnaires have been developed to assess hyperphagia in patients with PWS [15], only 2 have been developed to assess hyperphagia in patients and caregivers of patients with rare MC4R pathway diseases (Symptoms of Hyperphagia and Impacts of Hyperphagia questionnaires). While these questionnaires have not been used to determine treatment response in published interventional studies, they have been used to characterize the presentation and burden of hyperphagia in patients with BBS and their caregivers [19, 49]. Further, some assessments measure hunger alone but not hyperphagia, giving a partial view of the overall impact on hyperphagia symptoms (eg, food-seeking behaviors, hyperphagic drive). Of note, patients who are born with hyperphagia generally may not recognize satiety or be able to adequately describe normal hunger and are only able to do so after effective treatment [12, 14]. Further, patients with hyperphagia and cognitive impairment (eg, BBS, PWS) may not be able to provide self-reports and thus rely on caregiver-reported data [11]. These points highlight the utility of qualitative patient and caregiver interviews to establish a baseline or to determine subjective individual assessments of treatment efficacy and additionally suggest that treatment efficacy could be assessed in the context of withdrawal from intervention.

### Daily hunger questionnaire

Six publications reported the effect of setmelanotide on hunger in patients aged ≥ 12 years without cognitive impairment (ie, those who were able to self-report) as assessed by the Daily Hunger Questionnaire, which consists of 3 questions to determine daily average hunger (“In the last 24 hours, on average, how hungry did you feel?”), most hunger (“In the last 24 hours, how hungry did you feel when you were the most hungry?”), and morning hunger (“This morning when you woke up for the day, how hungry did you feel?”). Responses to each question are made on a Likert scale ranging from 0 (not hungry at all) to 10 (hungriest possible) [13, 71, 122–124, 126, 150–154]. Daily average, most, and morning hunger scores were averaged weekly for analysis [13, 124]. A psychometric evaluation estimated the threshold for meaningful within-patient change in daily most hunger score to be a reduction of ≥ 2 points [13]. Using this questionnaire, Phase 2 and 3 trials of patients with POMC deficiency (including biallelic variants in *POMC* or *PCSK1*); LEPR deficiency; BBS; HO; heterozygous *POMC*, *PCSK1*, or *LEPR* variants; steroid receptor coactivator 1 deficiency; SH2B adaptor protein 1 deficiency; or 16p11.2 chromosomal deletion have shown clinically meaningful hunger reduction with setmelanotide treatment (Table 1) [13, 71, 122–124, 126].

**Table 1:** ** Table** Pubications That Used Assessment Tools to Determine Hyperphagia Treatment Benefit

First author, year	Study design	Study population	N	Assessment tool	Hyperphagia-related outcome(s)
Argente, 2021 [122, 150]	Phase 2 uncontrolled trial of setmelanotide	Patients with obesity resulting from SH2B1 deficiency caused by an *SH2B1* variant or 16p11.2 deletion encompassing *SH2B1*	35	Daily Hunger Questionnaire	Mean percent change in most hunger score^a^ from baseline to Month 3 in patients aged ≥ 12 years was − 27.9% in weight responders (≥ 0.15 BMI Z score reduction [aged < 18 years] or ≥ 5% weight loss [aged ≥ 18 years]; n = 14) and − 25.8% in nonresponders (n = 18)
Clément, 2020 [124, 151, 152]	Phase 3, single-arm, open-label trial of setmelanotide with an 8-week double-blind, placebo-controlled withdrawal sequence	Patients with POMC deficiency caused by biallelic variants in *POMC* or *PCSK1* or LEPR deficiency caused by biallelic variants in *LEPR*	10 with POMC deficiency, 11 with LEPR deficiency	Daily Hunger Questionnaire	Mean percent change in most hunger score^a^ from baseline to ~ 1 year was − 27.1% (n = 7 aged ≥ 12 years) in patients with POMC deficiency and − 43.7% (n = 7 aged ≥ 12 years) in patients with LEPR deficiency
Ervin, 2023 [12]	In-depth interviews to qualitatively characterize the burden of hyperphagia and its modulation by setmelanotide	Patients and caregivers of patients with BBS who previously participated in Phase 2 and 3 trials of setmelanotide	19 (8 patients, 11 caregivers)	In-depth qualitative interviews	Peak hunger change with setmelanotide ranged from − 2 to − 6 points on a scale from 0 (not hungry at all) to 10 (hungriest possible); improved emotions, relationships, and ability to concentrate at work or school; decreased food-seeking behaviors
Farooqi, 2021a [123, 150]	Phase 2 uncontrolled trial of setmelanotide	Patients with obesity resulting from SRC1 deficiency caused by an *NCOA1* variant	30	Daily Hunger Questionnaire	Mean percent change in most hunger score^a^ from baseline to Month 3 in patients aged ≥ 12 years was − 28.9% in weight responders (≥ 0.15 BMI Z score reduction [aged < 18 years] or ≥ 5% weight loss [aged ≥ 18 years]; n = 9) and − 33.4% in nonresponders (n = 20)
Farooqi, 2021b [126, 150]	Phase 2 uncontrolled trial of setmelanotide	Patients with heterozygous variants in *POMC*, *PCSK1*, or *LEPR*	35	Daily Hunger Questionnaire	Mean change in most hunger score^a^ from baseline to Month 3 was − 4.4 points in weight responders (≥ 5% weight loss; n = 12) and − 2.3 points in nonresponders (n = 23)
Haqq, 2022 [13, 153]	Phase 3, 14-week, randomized, double-blind, placebo-controlled trial of setmelanotide followed by a 52-week open-label period	Patients with BBS or Alström syndrome	38	Daily Hunger Questionnaire	Mean percent change in most hunger score^a^ from baseline to Week 52 was − 30.9% in patients aged ≥ 12 years (n = 31); 62.5% of patients aged ≥ 12 years achieved ≥ 25% reduction in “most” hunger score
Roth, 2024 [71, 154]	Phase 2, open-label trial of setmelanotide	Patients with HO	18	Daily Hunger Questionnaire	Mean (percent) change in most hunger score^a^ from baseline to Week 16 in patients aged ≥ 12 years (n = 11) was − 2.9 points (− 45.0%)
Wabitsch, 2022 [14]	In-depth interviews to qualitatively characterize the burden of hyperphagia and its modulation by setmelanotide	Patients with POMC or LEPR deficiency who previously participated in Phase 2 and 3 trials of setmelanotide	3 with POMC deficiency, 2 with LEPR deficiency	In-depth qualitative interviews	Peak hunger change with setmelanotide ranged from − 1 to − 5 points on a scale of 0 to 10; improved emotions, relationships, and work or school performance; decreased food intake and improved satiety

^a^The most hunger score was captured daily using the question, “In the last 24 hours, how hungry did you feel when you were the most hungry?” Hunger was rated on a scale from 0 to 10 where 0 = “not hungry at all” and 10 = “hungriest possible.” Daily scores were averaged to calculate weekly scores for analysis. BBS, Bardet-Biedl syndrome; BMI, body mass index; HO, hypothalamic obesity; LEPR, leptin receptor; POMC, proopiomelanocortin; SH2B, Src homology 2B; SH2B1, SH2B adaptor protein 1; SRC1, steroid receptor coactivator 1

### Qualitative interviews

Two studies used in-depth qualitative interviews to characterize the presentation of hyperphagia and its modulation by setmelanotide in patients with POMC deficiency, LEPR deficiency, or BBS. Interviews followed a semistructured interview guide with open-ended questions about hunger, food-seeking behaviors, effects of hunger on various aspects of daily life, changes in these areas with setmelanotide, perceived meaningfulness of these changes, treatment satisfaction, and how participants would feel if they or the patient in their care needed to discontinue setmelanotide treatment [12, 14]. Both studies reported negative social and emotional consequences of hyperphagia that were alleviated with setmelanotide (Table 1), and qualitative data provided unique insight into the experiences of patients with rare MC4R pathway diseases and their caregivers that are, by nature, precluded by quantitative analysis [12, 14].

### Symptoms of hyperphagia and impacts of hyperphagia questionnaires

The Symptoms of Hyperphagia and Impacts of Hyperphagia questionnaires are described in detail elsewhere [15, 19, 49]. Briefly, these include 4 patient-reported or 5 caregiver-reported items that assess the frequency of food-seeking behaviors observed in the patients and 6 patient-reported or 10 caregiver-reported items that assess the extent to which the patient’s hunger or food-seeking behaviors impacted the patient’s and caregiver’s lives [19, 49]. In a psychometric evaluation of the Symptoms of Hyperphagia – Caregiver version and Impacts of Hyperphagia – Caregiver version in caregivers of patients with BBS, both instruments demonstrated reasonable reliability and validity and were determined acceptable for use in real-world settings [155]. If administered before and after an intervention, these questionnaires could provide a detailed understanding of the impact of hyperphagia management that spans a multifaceted view of quality of life.

### Ongoing clinical trials

There are currently 2 ongoing clinical trials that are using the Symptoms of Hyperphagia and Impacts of Hyperphagia questionnaires (Patient and Caregiver versions) to characterize hyperphagia before and after setmelanotide treatment in patients aged ≥ 6 years who have obesity caused by heterozygous variants in *POMC*, *PCSK1*, or *LEPR*; homozygous, heterozygous, or compound heterozygous variants in *NCOA1* or *SH2B1*; chromosomal 16p11.2 deletion encompassing *SH2B1*; or variants in ≥ 1 of 31 genes with strong or very strong relevance to the MC4R pathway [156–158]. These trials may enable future validation of the Symptoms of Hyperphagia and Impacts of Hyperphagia questionnaires in patients and caregivers of patients with rare MC4R pathway diseases other than BBS, thus meeting the critical unmet need for hyperphagia assessment tools appropriate for these patient populations [155]. Additionally, 10 clinical trials that used validated, indication-specific hyperphagia assessment tools to examine responses to treatment in patients with PWS were identified [159–168].

## Conclusions

Hyperphagia is a severe form of pathologic, insatiable hunger that confers multifaceted burden, with severe consequences experienced across multiple domains (eg, emotions, relationships, work, school) by both patients and caregivers of patients with rare MC4R pathway diseases [12, 14, 19, 28, 125]. Understanding the underlying cause of this condition is important for guiding effective personalized treatment approaches across individual patients and for highlighting the necessity of ongoing treatment, as current therapies are not curative. Advancements in the knowledge of the MC4R pathway and its role in regulating energy balance have led to the development of novel therapeutics that can provide targeted treatment options that directly address the underlying causes of hyperphagia. The appropriate assessment of the impact of treatment on hyperphagia symptoms can further inform efficacy in managing hunger. Despite this, there are currently no standard validated instruments for determining treatment benefit related to hyperphagia, which may be related to varied presentation across individuals [9, 12, 14, 29]. This work underscores the urgent need for consensus recommendations for indication-specific hyperphagia management in addition to validated instruments for testing efficacy of hyperphagia treatments [11]. Such developments may enable tailored management strategies that can alleviate the burden and HRQOL impacts of hyperphagia in the affected patient populations.

## Data Availability

No datasets were generated or analysed during the current study.

## Abbreviations

AgRP: Agouti-related peptide
ARC: Arcuate nucleus
BBS: Bardet-Biedl syndrome
BMI: Body mass index
fMRI: Functional magnetic resonance imaging
GABA: γ-Aminobutyric acid
GLP-1: Glucagon-like peptide-1
HO: Hypothalamic obesity
HRQOL: Health-related quality of life
LEPR: Leptin receptor
MBS: Metabolic and bariatric surgery
MC4R: Melanocortin-4 receptor
MSH: Melanocyte-stimulating hormone
NPY: Neuropeptide Y
PC1/3: Proprotein convertase 1/3
POMC: Proopiomelanocortin
PWS: Prader-Willi syndrome
QOL: Quality of life
RYGB: Roux-en-Y gastric bypass
SLR: Systematic literature review
WPAI: Work Productivity and Activity Impairment
